# Case report: Aortic valve endocarditis and recurrent pulmonary valve stenosis

**DOI:** 10.1186/s13019-023-02184-7

**Published:** 2023-03-21

**Authors:** Edina Korça, Gábor Veres, Gábor Szabó

**Affiliations:** Department of Cardiac Surgery, Middle German Heart Centre, University Hospital Halle (Saale), Martin-Luther University Halle-Wittenberg, Ernst-Grube-Str. 40, 06120 Halle (Saale), Germany

**Keywords:** Aortic valve, Pulmonary valve, Pulmonary stenosis, Endocarditis

## Abstract

**Background:**

We discuss a rare case of an adult patient with different pathologies involving the aortic and pulmonary valves in need of surgery.

**Case presentation:**

The patient had a history of congenital PV stenosis and surgical valvuloplasty. Almost 50 years later the patient underwent a complex second heart surgery due to infective endocarditis of the aortic valve and high-grade restenosis of the pulmonary valve. Replacement of the aortic and pulmonary valve, as well as reconstruction of the RVOT and closure of a persistent foramen ovale, followed. Postoperative course was uneventful and the patient was discharged home a week after surgery.

**Conclusion:**

Simultaneous surgery of pulmonary and aortic valves due to different pathologies is rare but can be performed successfully even in advanced age and can improve quality of life.

## Background

While aortic valve (AV) pathologies are mainly acquired, pulmonary valve (PV) pathologies are usually congenital.

However, simultaneous different diseases of the pulmonary and aortic valves in need of surgical treatment are uncommon. The only surgical procedure involving both of the above-mentioned valves is the Ross procedure in which the healthy autologous pulmonary valve is used to replace a diseased aortic valve in children. Even in this procedure only one of the valves is diseased. [1, 2]

We however present the surgical approach to a rare case of a patient with two different pathologies of these valves (aortic valve infective endocarditis (IE) and concomitant restenosis of the pulmonary valve).

## Case presentation

A 67-year-old patient was admitted to the emergency department with fever, vertigo, palpitations and a progressive feeling of weakness. Echocardiography showed a vegetation on the right coronary cusp (RCC) 0.3 × 0.6 cm. *Streptococcus sanguinis* was isolated in blood cultures. According to the Duke criteria, the diagnosis of IE was made. A tooth was considered as a possible focus of the infection and extracted during the hospitalization. Antibiotics were administered according to guidelines.

In the following echocardiographic examinations, no changes regarding the size of the vegetation were seen. Additionally, a high-grade stenosis with a middle-grade insufficiency of the PV [flow acceleration over the PV to just under 5.7 m/s (Max PG 128 mmHg)] and a moderately dilated right ventricle with an impaired systolic function were seen. Noteworthy was the patient’s history of congenital PV stenosis and surgical valvuloplasty in the seventies. Right heart catheterization revealed severe PV stenosis with a calculated valve opening area of 0.25 cm^2^ (peak-to-peak pressure gradient of 106 mmHg and a mean pressure gradient of 75.8 mmHg). Pulmonary hypertension was ruled out. Relevant stenosis of the coronary arteries was ruled out through coronarography. The decision for surgical treatment was made primarily due to high-grade PV restenosis.

**Fig. 1:**
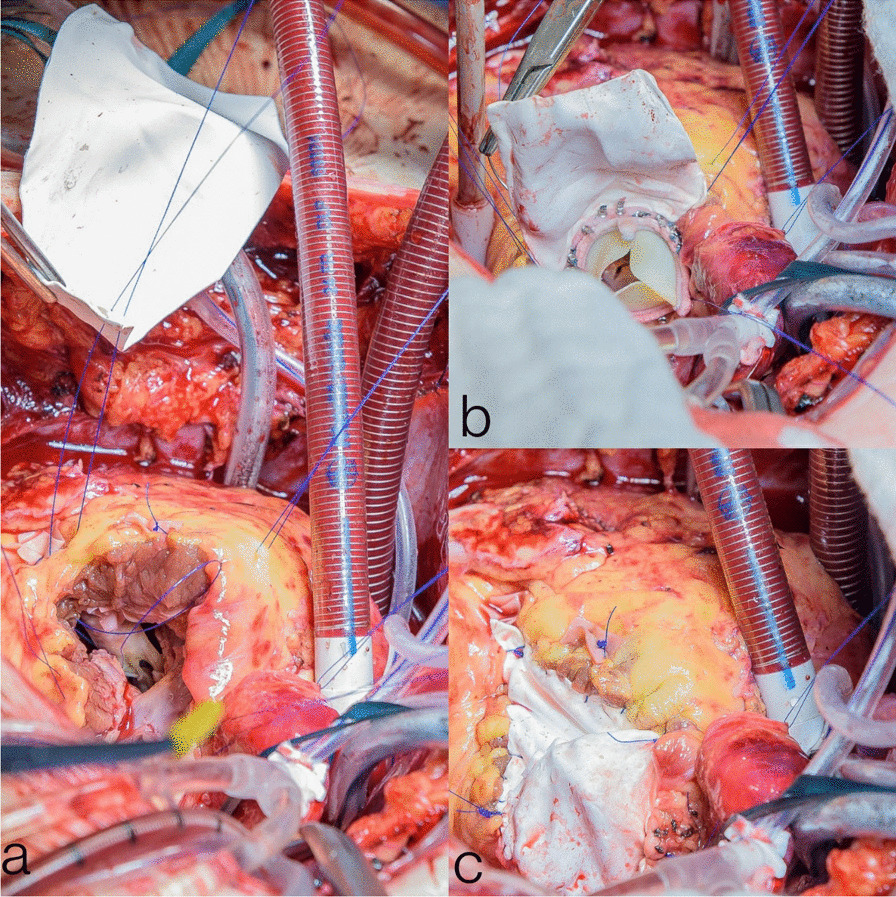
Intraoperative Pictures. **a** Gore-Tex-Patch was used to reconstruct the RVOT. **b** Gore-Tex-Patch and pulmonary valve prosthesis. **c** Final result

During the operation, severe adhesions as a consequence of pulmonary valve surgery in childhood were seen and detached. Following heparinization, the ascending aorta and both caval veins were cannulated. A vent catheter was inserted through the upper right pulmonary vein. Cooling of the patient to 34 °C followed. Two liters of Bretschneider solution were used for cardioplegia. Opening of the right atrium and direct closure of a persistent foramen ovale (5 × 5 mm) with subsequent closure of the right atrium followed. After aortotomy, multiple vegetations on the RCC were seen. The endocarditis-altered valve as well as a subvalvular membrane extending to the right and left coronary cusps were excised. A 21 mm CE Magna Ease valve was implanted and the aortotomy was closed. Next, following a longitudinal opening of the proximal pulmonary artery, the fibrotic altered valve with restricted mobility and a pronounced subvalvular hypertrophy with almost complete occlusion of the right ventricular outflow tract (RVOT) were seen. After a transannular extension to the right ventricle, of the initial longitudinal incision in the pulmonary artery, excision of the hypertrophied muscles and freeing of the cavity from trabecularized muscle parts followed. After sizing the RVOT using a 26 mm Hegar dilator, an 8 × 5 cm rhomboid Gore-Tex patch was sewn in with a continuous seam in the area of ​​the RVOT. Insertion of 18 sutures into the pulmonary valve annulus and the Gore-Tex patch with anchoring of a 23 mm CE Magna Ease prosthesis with the Cor-Knot system followed. At last, completion of the transannular patch enlargement in the area of the pulmonary artery with a continuous suture was done (Fig. 1). Weaning from the cardiopulmonary bypass and postoperative course were uneventful.

Subsequent TTE showed a good function of both prostheses. The patient was discharged from the hospital on the seventh postoperative day and antibiotics were administered remotely. A control TTE three months later showed a good function of both prosthetic valves and an improvement in the function of the right ventricle.

## Discussion

At the time of the patient’s first surgery in the seventies, balloon valvuloplasty was not yet available as a treatment option for PV stenosis. The first balloon valvuloplasty in humans was performed in 1982. Since then it has become the first choice of treatment in isolated valvular PV stenosis [3].

On the contrary pulmonary stenosis at different levels requires surgical treatment. Due to the necessity of early correction of congenital heart diseases, transannular patch plasty is more often performed in children. However, as described, it can be performed successfully even in adulthood, reducing heart load and improving quality of life. This technique can also be used in adults with infiltrative tumors of the right heart.

Even patients with corrected tetralogy of Fallot, in need of simultaneous reoperation on the aortic root and pulmonary valve are usually younger [4].

PV replacement with a homograft or biological valve is preferred due to a higher risk of thrombosis with mechanical valves on the PV position [5].

Follow-up studies have shown that while patients who have been treated for PV stenosis have a good outcome, 20% need a second intervention more than 20 years after the first one. Early treatment of patients with pulmonary stenosis has led to an increase in their life expectancy, it has also led to an increase in the number of patients who reach adulthood and need re-intervention [6]. Therefore, it is important to emphasize the need for regular check-ups to these patients, who consider themselves healed.

In our case, the patient did not have regular follow-ups after surgery and underwent a second intervention almost 50 years after the first one. A balloon valvuloplasty was not an option due to pulmonary stenosis at different levels as well as due to the coexistence of endocarditis of the aortic valve. Fetal endocarditis has been suggested as a possible etiology of pulmonary valve stenosis [2]. Studies show a possible genetic susceptibility to IE [7]. Moreau et al. showed a possible protective effect of SNPs on chromosome 3 against IE during Staph. aureus bacteremia [8]. Further studies on genetic susceptibility to endocarditis should be performed.

## Conclusion

Simultaneous surgery of pulmonary and aortic valves due to different pathologies is rare but can be performed successfully even in elderly patients and can improve quality of life. This case can serve as an additional reminder of how important good communication is, to raise the patient's adherence to therapy.

## Data Availability

Not applicable.
